# Status of the Mule Deer Population in Western Sonora, Mexico

**DOI:** 10.3390/ani16050725

**Published:** 2026-02-26

**Authors:** Juan Manuel Segundo-Galán, Enrique de Jesús Ruiz-Mondragón, Raul Valdez, Israel Guerrero-Cárdenas

**Affiliations:** 1 Independent Researcher, Hermosillo 83100, Mexico; manuel.segundo@yahoo.com.mx; 2 Laboratorio de Ecología, Centro de Investigaciones Biológicas del Noroeste, La Paz 23096, Mexico; guerrero04@cibnor.mx; 3 Fundación UABC, Santuario Cimarrón, Mexicali 21100, Mexico; 4 Department of Fish, Wildlife and Conservation Ecology, New Mexico State University, Las Cruces, NM 88046, USA; rvaldez@nmsu.edu

**Keywords:** aerial line transect, aerial survey, big game species, *Odocoileus hemionus*, wild ungulate population monitoring

## Abstract

The mule deer population in Mexico declined severely during the early 20th century but recovered due to public policies centered on sport hunting. Sonora is considered the main reference for mule deer management in Mexico; however, the conservation status of the species in the state remains unclear. This study aimed to estimate the relative abundance and population structure of mule deer in Sonora. An aerial survey was conducted from 4 to 24 November 2019 using linear transects distributed across 62 sampling quadrants (30 × 30 km each). Over 82 flight hours, a total of 1376 deer were observed, with a male:female:fawn ratio of 24:100:12. The number of sightings and deer observed per quadrant followed a normal distribution, with averages of five sightings and twenty-two deer per sampling unit. The quadrants with the lowest abundance corresponded to areas containing towns and agricultural fields, whereas the areas with the highest deer numbers coincided with those containing a high concentration of Wildlife Conservation Management Units, where habitat improvement actions were implemented. The results falls within the limits of a stable population of desert mule deer; however, the low proportion of fawns suggests that limiting factors may be exerting pressure on the population. These findings highlight the importance of continued habitat management and monitoring to promote population stability and support recruitment in Sonoran mule deer populations.

## Simple Summary

The conservation status of mule deer in the state of Sonora, Mexico, remains unclear because no large-scale monitoring of the species’ population has yet been carried out. This study estimated the relative abundance and population structure of mule deer in western Sonora. Aerial surveys were conducted from 4 to 24 November 2019, using linear transects distributed across 62 sampling quadrants (30 × 30 km each) in western Sonora. During 82 flight hours, a total of 1376 deer were observed, with a male:female:fawn ratio of 24:100:12. This is a ratio of age and sex classes that falls within the limits of a stable population of desert mule deer, although the low proportion of fawns suggests potential limitations on recruitment. On the other hand, the quadrants with the highest numbers of deer tended to coincide with areas containing a high concentration of Wildlife Conservation Management Units, where habitat improvement actions are implemented. The findings highlight the importance of continued habitat management and monitoring to support population stability and long-term conservation.

## 1. Introduction

In Mexico, the mule deer (*Odocoileus hemionus*) is distributed throughout the Chihuahuan and Sonoran deserts and along the entire Baja California Peninsula [1]. Although it is not classified within any risk category, it remains a conservation priority. This charismatic species requires extensive natural habitats to survive and reproduce and has significant ecological and social value [2]. It is an important source of protein for rural communities within its range, a valued game species, and plays a key role in ecosystem productivity [3].

Mule deer populations in Mexico were severely reduced in the early 20th century due to intensive commercial hunting driven by the growing demand for meat in border cities [4]. However, populations began to recover following the implementation of hunting regulations, the commercialization of sport-hunting permits, and the translocation of individuals from the southern United States [5,6]. Furthermore, in the northern states of Mexico, the conservation of the mule deer, like that of other wildlife, was aided by the transition of land ownership from ejidos (communally owned and managed lands under a system supported by the federal government) to the private sector further strengthened protections against poaching [7,8]. Regarding this last point, it is important to acknowledge that while the shift from communal to private property had a positive effect on wildlife conservation, it also had significant social implications, resulting in cultural erosion and the loss of traditional knowledge within rural Mexican communities [9].

The state of Sonora is a model in the management of mule deer in Mexico. Landowners prioritized big game and reduced or removed cattle from the species’ habitat [5]. In addition, they took a number of measures to boost the deer population on their land: artificial water and food supplementation, re-establishment of native shrubs and control of undesirable brush species [10,11]. These management actions have impacted the conservation of the species, producing resilient deer populations that generate significant economic revenue for the state through the sport hunting. This activity has become a major source of income for the rural sector [12]: during the 2022–23 hunting season, 3429 hunting permits for mule deer were sold [13] at a minimum of US$ 5500 each [14], generating US$ 18,859,500 in revenue in Sonora alone.

Although Sonora is considered a model region for mule deer management in Mexico [5], few studies have been conducted on the species [15]. Therefore, key aspects of the biology and ecology of mule deer in the state remain poorly understood [16]. One of the most critical knowledge gaps concerns the species’ abundance and population structure, as no regional or statewide monitoring programs have been implemented to date. Efforts to compile data from population surveys in the state’s wildlife management units have been limited, and such estimates are unreliable due to inconsistent sampling methods [17,18]. Consequently, in 2019, the Technical Advisory Council on Wildlife of Sonora recommended conducting an aerial survey to assess the species’ population status in western Sonora [19]. This area is within the Southwest Deserts Ecoregion which encompasses the Mojave, Sonoran and Chihuahua deserts [20].

Population monitoring is a fundamental tool for collecting the biological and systematic data needed to assess the conservation status and demographic trends of wildlife, develop management plans and determine hunting quotas [21,22]. It also allows for evaluating the factors affecting populations and the effectiveness of conservation measures [23]. For mule deer, information on conservation status is especially important, as populations fluctuate frequently, particularly in arid ecosystems where climatic conditions and local habitat characteristics strongly influence these dynamics [24].

Aerial monitoring by helicopter is a primary method for assessing populations of large mammals over extensive geographic areas, as it is considered the most reliable approach [25,26]. This technique allows for the observation of wildlife over extensive areas, provides clear visibility, and facilitates the determination of the population structure of ungulates. It also enables coverage of diverse habitats, including valleys, mountain ranges, scrublands, and forests [27,28]. For mule deer, aerial surveys have proven to be the most accurate method for estimating population abundance, structure, and density [29].

In this context, the objective of the study was to estimate the relative abundance and population structure of mule deer in Sonora. The information obtained will provide a baseline for evaluating the effectiveness of ongoing conservation efforts in the state.

## 2. Materials and Methods

### 2.1. Study Area

**Figure 1:**
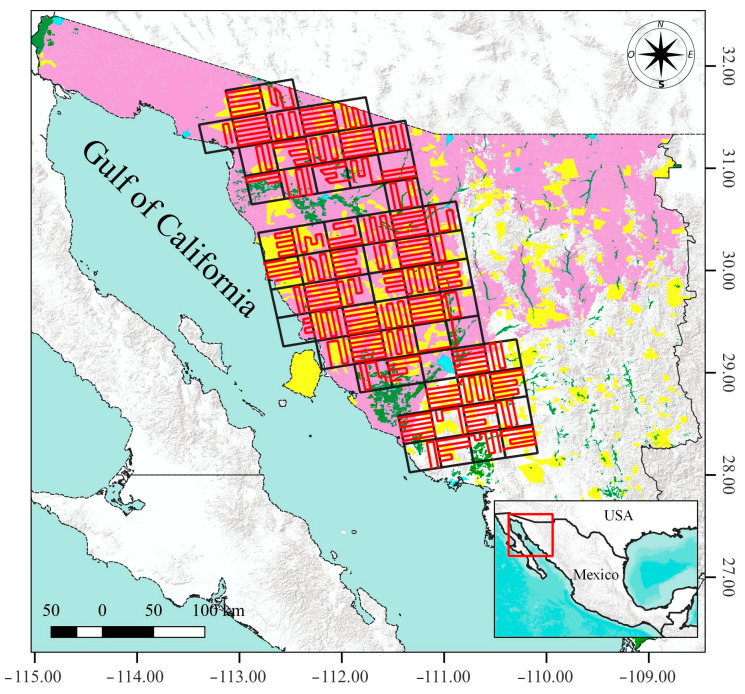
Study area and sampling design for the Sonoran mule deer population survey. The map shows sampling quadrants (grid), aerial survey transects (red lines), mule deer range (purple), Wildlife Conservation Management Units (UMA) in Sonora (yellow), agricultural fields (green), settlements (blue), Mexican state boundaries (black lines), and topography (gray).

The study area, located in western Sonora between 113°16′52″ W/32°01′34″ N and 109°58′53″ W/28°03′56″ N (Figure 1) comprises a mosaic of hills, rugged mountain ranges, and plains [30]. The region has an arid, semi-warm climate, with an average annual rainfall of 100–300 mm [31,32], and is dominated by microphyll desert scrub and sarcocaule scrub [33]. This is one of the main livestock producing areas in Sonora, where it is estimated that there are around 400,000 head of cattle [34].

### 2.2. Aerial Survey

The monitoring method employed was linear aerial transects [35]. The study area was divided into 30 × 30 km quadrants, which covered the entire range of the mule deer [36]. Quadrants containing cities, or where agricultural fields covered more than 50% of the area, were eliminated. Similarly, Wildlife Conservation Management Units (UMA) for mule deer were used to define the northern and southern boundaries of the grid.

A total of 62 sampling units were defined, covering an area of 5580 km^2^ of mule deer habitat (Figure 1). Surveys were conducted along alternating longitudinal and latitudinal transects, each separated by 5 km to ensure independence of observations. Some transects were adjusted to avoid towns, agricultural fields, and mountainous areas. These latter sites were avoided because the terrain prevents low-altitude, linear aerial transects from being flown.

Monitoring was conducted from 4 to 24 November 2019 using a five-seat Bell 505 helicopter. The team consisted of three observers and a pilot, all experienced in aerial surveys of mule deer in Mexico. The aircraft traveled at an average speed of 100 km/h and an average altitude of 25 m above ground level [29]. For mule deer in a desert ecosystem, at this speed and altitude, the width of the field of vision is 100 m on either side of the transect, and the detectability index ranges from 26% to 58% [37]. Upon sighting a deer or a herd, the team recorded the geographic location and number of individuals, classifying them by age and sex (male, female, or fawn).

## 3. Results

During 82 h of effective flight, an area of 1686 km^2^ was surveyed (covering 8429 km in a straight line with a viewing width of 0.2 km). During this period, 311 mule deer sightings were recorded, including both individual and group observations (Table 1). A total of 1376 individuals were observed, comprising 246 males, 1011 females, and 119 fawns. This corresponds to a male:female:fawn ratio of 24:100:12. Based on the species’ detectability index for desert ecosystems, the estimated population abundance in the sampled area (1686 km^2^) ranges from 2372 to 5292 individuals.

**Figure 2:**
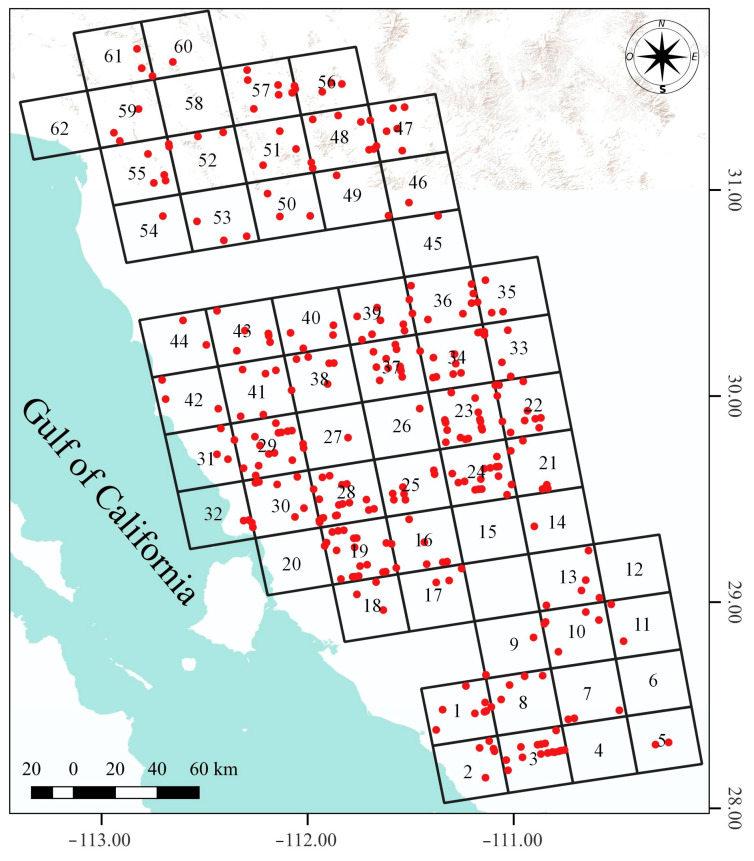
Spatial distribution of mule deer sightings (red dots). Sampling quadrants are shown, each labeled with its corresponding number.

The number of deer sightings per sampling unit followed a normal distribution (*p* > 0.01 Shapiro–Wilk), with an average of five observations per quadrant. However, the species was not detected in all sampling units. Mule deer were not observed in quadrants 4, 6, 12, 15, 20, 58, and 62. Conversely, quadrants with counts of sightings > 1 SD were 34, and 23 with 14 records, and with >2 SD were 19, 28, 24, 30, 3 with 21, 18, 16, 16, and 15 respectively (Figure 2).

The numbers of males, females, and fawns, as well as the total number of deer observed in each quadrant, also followed normal distributions (*p* > 0.01 Shapiro–Wilk). On average, there were four males, sixteen females, two fawns, and twenty-two individuals per quadrant. Highest counts of males (>1 SD) were recorded in quadrants 19, 36, 24, 30, 48, 28, and 3, with 20, 18, 16, 16, 15, 14, and 12 individuals, respectively. Highest counts of females (>1 SD) were recorded in quadrants 23, 48, 3, 19, 34, 24, and 57, with 82, 81, 62, 55, 54, 54, and 44 individuals, respectively. Highest counts for fawns (>1 SD) were recorded in quadrants 3, 2, and 1, with 11, 9, and 7 individuals, respectively. Similarly, the highest total deer counts (>1 SD) were recorded in quadrants 48, 23, 3, 19, 24, and 34, with 101, 98, 85, 79, 75, and 63 individuals, respectively (Table 1). Males, females, and fawns were not observed in all quadrants. Males were absent from ten quadrants (7, 26, 33, 41, 42, 45, 46, 53, 54, and 55), females were not observed in three quadrants (14, 27, and 60), and no fawns were sighted in eighteen quadrants (9, 11, 14, 16, 26, 27, 40, 42, 44, 45, 46, 47, 51, 52, 53, 54, 55, and 60).

**Figure 3:**
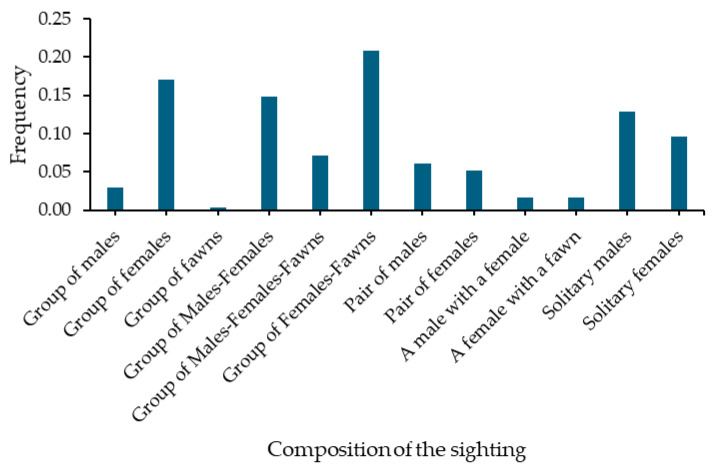
Composition of sightings from aerial monitoring of the western Sonora mule deer population.

Of the 311 mule deer sightings, 23% (70) were solitary individuals, 14% (45) were pairs, and 63% (196) were groups of three or more. Groups (≥3 deer) composed of females with fawns, females only, males with females, males and females with fawns, males only, and fawns only were observed. In addition, pairs of males, pairs of females, male–female pairs, and female–fawn pairs were observed. Solitary males and solitary females were also observed (Figure 3). The groups consisted of an average of 4 ± 4 individuals, ranging from 3 to 50 deer. Outlier groups (>1 SD) included 13, 17, 18, 20, 22, and 50 individual deer.

## 4. Discussion

The minimum population size of mule deer in western Sonora was between 2372 and 5292 individuals. This estimate corresponds only to the sampled area of 1686 km^2^, which represented 30% of the total study area. The wide range in the estimate results from several factors that introduce negative biases in aerial counts [38]. In desert ecosystems, the detectability of mule deer typically ranges from 26% to 58%, as the likelihood of detecting individuals decreases with increasing perpendicular distance from the transect, shrub cover, light brightness, and terrain roughness. Detectability is also influenced by animal behavior, since the probability of observation increases substantially when individuals are in motion [37].

The male:female ratio in mule deer populations inhabiting desert ecosystems ranges from 9:100 to 63:100 [39,40]. The ratio recorded in Sonora (24:100) falls near the midpoint of this range. This proportion is considered acceptable for a population subject to extractive management and is even higher than ratios reported for some populations in forested habitats, where only 10 to 24 males are found for every 100 females [41].

On the other hand, the female:fawn ratio in mule deer populations inhabiting arid regions ranges from 100:10 to 100:85 [39,40]. A ratio of ≤100:15 is considered low and is typically associated with limited rainfall and reduced forage availability [40]. However, low ratios may also be influenced by predation, which has been reported to cause mortality of up to 64% of fawns in desert mule deer populations [42]. In this context, the number of fawns per 100 females observed in Sonora (100:12) can therefore be considered low.

The absence of mule deer sightings in a quadrant does not imply the absence of the species in that sampling unit. As mentioned above, various factors affect the detection of deer, and the flight only covered a fraction of the area. However, the sampling units in which no deer were observed have characteristics that limit the species’ presence. Quadrants 4, 12, 15, and 58 contain towns and agricultural fields, environments that restrict the distribution of these animals [43,44,45]. Meanwhile, the topography and type of vegetation in quadrants 6, 20, and 22 are not ideal for mule deer. In the first two quadrants, the terrain is rugged, and in the third, sandy desert vegetation dominates [30,32,46].

The sampling units with the highest number of sightings and individuals are located far from population centers and do not contain agricultural fields [33]. They also include several UMA where habitat improvement actions have been implemented to attract and retain mule deer. These actions include artificial food and water supplementation, transplanting browse species, shredding for brittlebush control in buffelgrass pastures, improving native rangelands infested with brush by disk harrowing, and adopting grazing systems [10,11,47]. The results of the aerial survey indicate that these habitat improvement actions were associated with higher observed abundance of mule deer population. Quadrants 3, 19, 24, and 48 are particularly noteworthy, as they exhibited the highest numbers of both males and females, as well as total individuals.

The absence of males, females, and fawns in certain sampling units is due to low population density. In most sampling units where no age or sex was observed, only one to three sightings of the species were documented. Quadrant 41 is notable because five groups of mule deer were observed there (Figure 2), exceeding the average number of sightings per quadrant ($\overset{-}{x}$ = 3); however, none of these groups included a male. Interestingly, a high number of males were observed in an adjacent quadrant (quadrant 30), so it is possible that at the time of the aerial survey, most of the males in this region were concentrated in that sampling unit.

The results regarding group size are consistent with typical mule deer populations in desert ecosystems, where group sizes generally average five individuals and range from one to 24 [39]. Similarly, the composition of the groups was consistent with what would be expected for a population of mule deer prior to the rut season in a desert ecosystem. Most males were observed in male-only groups or alone, while groups containing both males and females were low (<26%) [39]. Notably, the most observed group type was females with fawns, even though fawns were not abundant in the study. This was because these groups consisted of many females (between two and twelve) and a small number of fawns (between one and eight).

## 5. Conclusions

In 2019, the minimum population size of mule deer in western Sonora was estimated to be between 2372 and 5292 individual deer, with a ratio of males to females to fawns of 24:100:12. These results fall within the limits of a stable desert mule deer population. However, the relatively low proportion of fawns may suggest limitations in recruitment due to environmental stressors, such as drought. Meanwhile, the high proportion of males in a hunted population indicates that hunting is well managed in the region. On the other hand, no deer were observed in some sampling units that included towns, agricultural fields, rugged terrain, and sandy desert vegetation. The quadrants with the highest deer numbers coincided with those containing a high concentration of UMA, where habitat improvement actions were implemented. Habitat management measures implemented in the UMA located in quadrants 48, 23, 3, 19, 24, and 34 appear effective in enhancing mule deer abundance. On average, the deer groups consisted of five individual deer, ranging from one to 24. Since monitoring occurred prior to the rut season, most males were separated from females. This is the first study to report on the abundance and population structure of mule deer in western Sonora, and it will therefore serve as a benchmark for assessing the conservation status of the species in the region and determining the success of implemented management policies, plans, and programs.

## Figures and Tables

**Table 1:** Summary of aerial survey results for the Sonoran mule deer population. The dash indicates that there were no observations.

Quadrant	Sighting	Male	Female	Fawn	Total Deer	Quadrant	Sighting	Male	Female	Fawn	Total Deer
1	8	3	28	7	38	32	5	2	12	2	16
2	6	4	17	9	30	33	3	—	16	3	19
3	15	12	62	11	85	34	14	3	54	6	63
4	—	—	—	—	—	35	4	2	12	6	20
5	2	3	10	4	17	36	9	18	26	1	45
6	—	—	—	—	—	37	11	3	41	4	48
7	3	—	7	2	9	38	6	3	14	5	22
8	5	4	4	1	9	39	7	9	10	2	21
9	3	3	7	—	10	40	4	5	3	—	8
10	3	1	11	1	13	41	5	—	25	2	27
11	2	2	10	—	12	42	3	—	6	—	6
12	—	—	—	—	—	43	6	3	11	2	16
13	5	3	19	3	25	44	2	1	3	—	4
14	1	2	—	—	2	45	1	—	3	—	3
15	—	—	—	—	—	46	1	—	1	—	1
16	5	1	14	—	15	47	5	8	18	—	26
17	3	4	4	2	10	48	9	15	81	5	101
18	3	1	9	2	12	49	2	3	2	1	6
19	21	20	55	4	79	50	3	1	9	1	11
20	—	—	—	—	—	51	3	2	9	—	11
21	5	4	30	2	36	52	4	4	3	—	7
22	11	5	30	5	40	53	3	—	14	—	14
23	14	10	82	6	98	54	1	—	1	—	1
24	16	16	54	5	75	55	4	—	11	1	12
25	2	4	5	1	10	56	3	4	12	2	18
26	1	—	5	—	5	57	8	10	44	1	55
27	1	2	—	—	2	58	—	—	—	—	—
28	18	14	30	1	45	59	3	3	15	—	18
29	6	5	14	2	21	60	1	1	—	—	1
30	16	16	33	5	54	61	3	4	8	1	13
31	3	3	7	1	11	62	—	—	—	—	—
Totals		Sightings = 311			Males = 246		Females = 1011			Fawns = 119	

## Data Availability

The data presented in this study are available on request from the corresponding author.
